# Analysis of Fluctuation in the Heme-Binding Pocket and Heme Distortion in Hemoglobin and Myoglobin

**DOI:** 10.3390/life12020210

**Published:** 2022-01-29

**Authors:** Hiroko X. Kondo, Yu Takano

**Affiliations:** 1 Faculty of Engineering, Kitami Institute of Technology, School of Regional Innovation and Social Design Engineering, 165 Koen-cho, Kitami 090-8507, Japan; 2 Graduate School of Information Sciences, Hiroshima City University, 3-4-1 Ozukahigashi Asaminamiku, Hiroshima 731-3194, Japan; ytakano@hiroshima-cu.ac.jp; 3 Laboratory for Computational Molecular Design, RIKEN Center for Biosystems Dynamics Research, 6-2-3, Furuedai, Suita 565-0874, Japan

**Keywords:** heme distortion, pocket rigidity, protein environment, hemoglobin, myoglobin, MD simulation, ONIOM

## Abstract

Heme is located in the active site of proteins and has diverse and important biological functions, such as electron transfer and oxygen transport and/or storage. The distortion of heme porphyrin is considered an important factor for the diverse functions of heme because it correlates with the physical properties of heme, such as oxygen affinity and redox potential. Therefore, clarification of the relationship between heme distortion and the protein environment is crucial in protein science. Here, we analyzed the fluctuation in heme distortion in the protein environment for hemoglobin and myoglobin using molecular dynamics (MD) simulations and quantum mechanical (QM) calculations as well as statistical analysis of the protein structures of hemoglobin and myoglobin stored in Protein Data Bank. Our computation and statistical analysis showed that the protein environment for hemoglobin and myoglobin prominently affects the doming distortion of heme porphyrin, which correlates with its oxygen affinity, and that the magnitude of distortion is different between hemoglobin and myoglobin. These results suggest that heme distortion is affected by its protein environment and fluctuates around its fitted conformation, leading to physical properties that are appropriate for protein functions.

## 1. Introduction

Heme is a complex of iron and porphyrin that is present at the active site of heme proteins. Heme proteins, a group of proteins that bind heme(s) as a cofactor, perform diverse biological functions, such as oxygen binding (transport or storage) [1,2], electron transfer [3], redox reactions [4], microRNA processing [5], and transcriptional regulation [6,7], indicating that heme molecules have diverse functions. Heme is classified according to the nature of its peripheral groups. The common types of heme are heme *b* and heme *c*, and other important types are heme *a* and heme *o*. Although the functional mechanisms of individual heme proteins have been thoroughly studied, elucidating the mechanisms that regulate the diverse functions of heme is still challenging. To solve this issue, we have been investigating the structure–function relationship in heme proteins by focusing on the distortion of heme porphyrin. Heme distortion has recently been considered an important factor underlying the diverse functions of heme, because it correlates with the physical properties of heme, such as oxygen affinity and redox potential [8,9,10,11]. Bikiel et al. examined the effect of the distortion on the binding affinity of oxygen molecule and showed that out-of-plane distortions tend to decrease the oxygen affinity, while the in-plane distortions increase or decrease it [8]. Imada et al. conducted the systematic study about the relationship between the heme distortion (saddling and ruffling distortions) and redox potential and indicated that the ruffling distortion tends to decrease the redox potential, while the saddling distortion has a tendency to increase it [9]. Kanematsu et al. analyzed the difference in molecular structure of heme between the protein groups of oxidoreductase and oxygen-binding protein and succeeded to extract a feature that correlates with both the redox potential and oxygen affinity [10]. Sun et al. studied cytochrome c_551_ and its mutant with different magnitudes of the ruffling distortion and showed that the ruffling distortion is correlated with the electron transfer rates, suggesting that the ruffling distortion could play a significant role in redox control [11]. One of our interests is the structural regulation of heme by its host protein environment, and our recent study suggests a correlation between the amino acid composition of the heme-binding pocket and particular heme distortions [12]. According to a survey of the structures of heme proteins stored in the Protein Data Bank (PDB; http://www.rcsb.org/, accessed on 27 April 2020) [13], hemes in proteins with the same amino acid sequence have similar conformations.

Some studies have reported a correlation between the protein environment and heme distortion, suggesting that the protein environment of the heme-binding pocket is probably rigid and affects heme distortion. Li et al. demonstrated the stability of the heme-binding pocket by comparing protein structures in the apo (heme-unbound) and holo (heme-bound) states [14]. Most apo–holo pairs exhibit a small conformational change after heme binding (root mean square deviation of 1.03 Å or less). Furthermore, Sacquin-Mora et al. investigated the local flexibility of the active-site residues using Brownian dynamic simulations and showed that the amino acid residues around the heme-binding pocket must be tightly anchored to enable its biological function, except for the residues that are mobile according to their protein function [15]. We thus investigated the fluctuation of the heme-binding pocket and heme distortion in the host protein environment and the correlation between them.

We focused on two heme proteins, hemoglobin (Hb) and myoglobin (Mb). Although both have oxygen-binding activities, their functions are different. Whereas Mb is a monomeric protein that stores oxygen and facilitates its diffusion in muscle, Hb is a tetramer protein that transports oxygen in the blood. Mb and Hb tertiary structures were determined by the earliest crystallographic studies [1,2], and their functional mechanisms have been thoroughly investigated. Even in recent years, some studies have focused on their dynamics. Aharoni and Tobi compared the dynamics of Mb and Hb using algorithms for alignment of Anisotropic Network Model modes of motion and indicated that the quaternary structure of Hb affects the intrinsic dynamics of each domain, leading to functional differences between Mb and Hb [16]. Tobi also carried out the clustering of globin family proteins based on their dynamics and succeeded in distinguishing the different states of Hb [17]. Bringas et al. elucidated a mechanism that regulates oxygen affinity through structural changes in Hb subunits using molecular dynamics (MD) simulations and quantum mechanics (QM)/molecular mechanics (MM) calculations [18]. Based on these meaningful results, it is important to elucidate the effect of these differences in protein dynamics on heme distortion.

In this study, we first estimated the effect of ligand(s) on heme distortion for an isolated heme via QM calculations and then compared heme distortion between an isolated heme and hemes in Hb and Mb based on PDB data. Next, to clarify the relationship between the protein environment and heme distortion, we investigated the fluctuation in the heme-binding pocket and heme distortion in its host protein for Hb and Mb using MD simulations and QM calculations. The results showed differences in flexibility of heme-binding pockets and in the tendency of fluctuations in the doming distortion of heme between Hb and Mb, with the latter corresponding to the results obtained from the PDB survey. These differences might be related to differences in function between Hb and Mb.

## 2. Materials and Methods

### 2.1. Collation of Structural Data of Hemoglobin and Myoglobin

**Figure 1:**
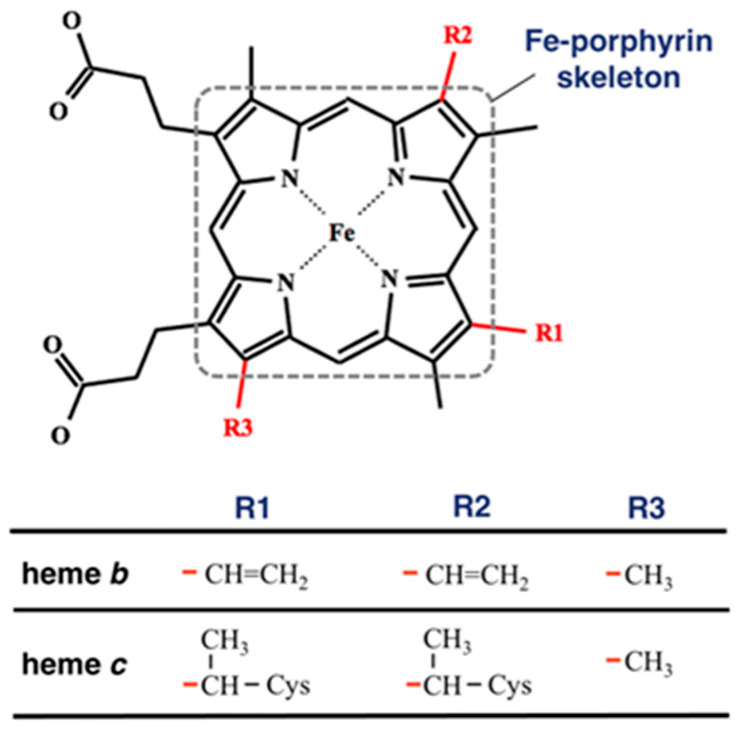
Structure of heme *b* and heme *c*. The red lines in the bottom table correspond to the bonds in the structure of the heme molecule. The atoms composing the Fe-porphyrin skeleton are surrounded by a square with a dotted line.

To compare the heme distortions in hemoglobin and myoglobin, first, we downloaded PDBx/mmCIF and fasta files of any kinds of heme proteins carrying heme *b* and heme *c* (Figure 1) with a structural resolution less than or equal to 2.0 Å from PDBj [19] and extracted structural information from them. This is because hemoglobin and myoglobin contain heme *b* or heme *c*. The compound ID (_chem_comp.id) HEM, HEB, or HEC was used to search for heme proteins via SQL in the PDBj Mine relational database (https://pdbj.org/mine, accessed on 22 December 2021) [20]. Consequently, 3070 unique entries were selected from all PDB entries (as of 22 December 2021). For each entry, the structural data of heme without missing data of the atomic coordinates of the 25 heavy atoms composing the Fe-porphyrin skeleton, shown in Figure 1, were extracted. We obtained 3063 entries (list of PDB IDs is available in Supplementary Materials). The Bio.PDB package [21] in Biopython library [22] was used as a parser and structural data were collected using the MDTraj library [23]. Next, we carried out the clustering for amino acid sequences for collated entries using Cd-Hit [24] with a clustering threshold of a sequence identity of 90% and obtained the clusters of PDB chains of the α chain of hemoglobin, the β chain of hemoglobin, or myoglobin. The cluster of the α chain of hemoglobin has 235 chains, that of the β chain of hemoglobin has 287 chains, and that of myoglobin has 279 chains.

Heme porphyrin distortion was analyzed using normal-coordinate structural decomposition (NSD) [25], which is a typical approach for evaluating heme distortion. NSD is a method that represents the porphyrin distortion as linear combinations of distortions along the vibrational modes. The equilibrium structure and vibrational modes of heme were calculated by using the PBE0 hybrid functional [26] with the 6–31G(d) basis sets [27,28,29]. Only the saddling, ruffling, and doming modes were considered in this study. The details of the calculation were described in a previous study [30].

### 2.2. Setup of Simulation Systems

In this study, we considered six systems, deoxy-Mb (dMb), oxy-Mb (oMb), apo-Mb (apoMb), deoxy-Hb (dHb), oxy-Hb (oHb), and apo-Hb (apoHb). The initial structures of the simulations were prepared using the atomic coordinates determined by X-ray crystallographic studies (PDB ID: 1A3N [31] (human) at 1.8 Å resolution for dHb and apoHb, 2DN1 [32] (human) at 1.25 Å resolution for oHb, 1MBD (sperm whale) at 1.4 Å resolution for dMb and apoMb, and 1MBO [33] (sperm whale) at 1.6 Å resolution for oMb). The initial structures of an apo protein were prepared by removing the heme molecule(s) from its complex structure in the deoxy state. All ionizable side chains were configured in their ionized states at pH 7.0, as calculated using the H++ server [34], except for 146His in oHb. Because protonation of the C-terminal histidines of Hb β chains, namely 146His (HIP), is an important factor for stabilizing the quaternary T state [35,36], only the Nε atom of 146His was protonated in the oxygenated state. Each system was solvated with a 150 mM NaCl aqueous solution in a rectangular box. The distance between the solute and box was set to 10 Å.

### 2.3. Simulation Details

All simulations were conducted using GROMACS software, version 2019.5 [37]. The Amber ff14SB force field [38] was applied to normal amino acids and ions, and the TIP3P model [39] was applied to water molecules. For heme molecules, we used the force field according to Bringas et al. [18]. The periodic boundary condition was applied, and electrostatic interactions were treated using the Particle Mesh Ewald method [40]. The cutoff distances for both the Ewald real space and van der Waals truncation were 12 Å. The neighbor list was updated every 20 steps for MD simulations. The simulation protocols were the same for all systems.

After the energy minimizations on the whole system, we gradually heated the system from 0.5 K to 300 K for 120 ps (50 K/20 ps) using a V-rescale thermostat [41]. Subsequently, two equilibration processes with the position restraints of the heavy atoms of the proteins were applied to relax the hydrogen atoms as follows: 100 ps NVT (constant volume and constant temperature) simulation with force constants of 2.5 kJ mol^−1^ Å^−2^ for backbone atoms, 0.5 kJ mol^−1^ Å^−2^ for sidechain atoms, and 200 ps NPT (constant pressure and constant temperature) simulation with force constants of 0.5 kJ mol^−1^ Å^−2^ for backbone atoms. We performed each 200 ns production run under NPT conditions, with a time step of 2 fs. The temperature and pressure were maintained at 300 K and 1 bar with a Nosé–Hoover thermostat [42,43,44] and Parrinello–Rahman barostat [45,46], respectively. All bonds involving hydrogen atoms were constrained using the LINCS algorithm [47]. For the simulation of Mb (dMb and oMb), a distance restraint was applied between the Fe atom of heme and the nitrogen atom (NE2) of the ligand histidine with a harmonic potential with a force constant of 10 kJ mol^−1^ Å^−2^, because the coordination between heme and the ligand histidine could not be maintained without restraints. The reference distance was set as the coordination distance in the X-ray structure as follows: 2.096 Å for dMb, and 2.065 Å for oMb. No restraint was applied for Hb simulations. Two simulations with different initial velocities were conducted for each system.

### 2.4. Trajectory Analysis of MD Simulations

Snapshots were sampled every 20 ps for each trajectory. In the trajectory analyses, we defined “pocket residues” as amino acid residues for which heavy atoms are within 4.5 Å of the Fe-porphyrin skeleton of the heme molecule in the energy-minimized structure (reference structure in the following section). The structural stability of the whole structure and pocket residues was estimated using root mean square deviation (RMSD) values from the reference structure. RMSD values were computed for Cα atoms after superimposing each region (whole structure or pocket residues) onto the reference structure. The root mean square fluctuation (RMSF) values were calculated for each heavy atom after least-squares fitting of the Ca atoms of the whole protein and averaged over the atoms in each residue. The MDTraj library [23] was used for the analyses.

For the analysis of volume and clustering of the heme-binding pocket, snapshots were sampled every 5 ns for each protein subunit. The pocket volume was computed using POVME 3.0 [48]. The sampled structures (heme–protein complex) were superimposed by Fe and the four nitrogen atoms in heme. The volume was calculated using the following parameters: the center of the mean coordinates of the Fe atom of heme as the center of an inclusion sphere and 8.5 Å as the radius of an inclusion sphere. This is because the heme is fitted into a sphere with a radius of 8.5 Å centering around Fe atom (the distances between Fe and an oxygen atom of propionates were approximately 8.5 Å). Hierarchical clustering of the heme-binding pocket was also performed using POVME 3.0 with the results obtained from the volume calculation, and the representative structures of each cluster were obtained.

### 2.5. Model Construction for QM Calculations

In this study, we used three types of models as follows: the QM model of isolated heme (1), our own N-layered integrated molecular orbital and molecular mechanics (ONIOM) model of heme complexed with its host protein (2), and the QM model of heme and adjacent residues (3). (1) A model of isolated heme with or without ligands consists of heme or heme and its ligand(s). The initial coordinates were extracted from the X-ray structures of deoxygenated and oxygenated hemoglobin (PDB IDs: 1A3N and 2DN1, respectively). (2) The ONIOM model includes heme and its host protein subunit, for example, an Hb chain. The high layer consists of heme and its ligand(s), and the Cα atom of the ligand, histidine, is replaced with a hydrogen atom. (3) The QM model of heme and adjacent residues is composed of heme and amino acid residues, for which heavy atoms are within 4.5 Å of the Fe-porphyrin skeleton of heme. The backbone atoms of the amino acid residues were removed, and the Cα atoms were replaced with hydrogen atoms. X-ray structures (PDB ID: 1MBD:A, 1MBO:A, and 1A3N:A) were used as the initial structures. Each model was illustrated in Figure S1.

### 2.6. Estimation of Heme Porphyrin Distortion Based on QM Calculations

We performed geometry optimizations on models (1) to (3) in the aforementioned section using the PBE0 hybrid function [26] with the 6–31G(d) basis set [27,28,29]. For model (1), the coordinates of all atoms were optimized, and the protein environment or water solvent was included using the polarizable continuum model (PCM) [49,50,51] with a dielectric constant of 4.0 or 78.4, respectively. For model (2), only the coordinates of heme and its ligand(s) were optimized (backbone atoms except the Cα atom of the ligand histidine were fixed). An AMBER force field was used for the low (molecular mechanics) layer. For model (3), only the coordinates of the heme, oxygen atoms in the O_2_ molecule, and hydrogen atoms in the amino acid residues were optimized. The six dihedral angles involved in the propionates were fixed during optimization, and the protein environment was included using PCM with a dielectric constant of 4.0. All QM calculations were performed using the Gaussian16 program package [52].

## 3. Results and Discussion

### 3.1. Distortion of Heme Porphyrin in Isolated Heme

Bikiel et al. [8] analyzed the distortion of the Fe-porphyrin molecule (heme without side-chains) in the five-coordinate state (mono His-coordinated) and six-coordinate state (His-O_2_-coordinated) through DFT calculations and showed that the doming distortion tends to decrease from the five-coordinate state to the six-coordinate state. In this study, to examine the effect of ligand(s) on its distortions in heme *b* (the chemical structure is presented in Figure 1), we computed the porphyrin distortion of heme *b* in the mono His-coordinate and His-O_2_ coordinate states. The coordinates of all atoms were optimized (no atom was fixed) by PBE0/6–31G(d) with the PCM with dielectric constants of 4.0 and 78.4 to incorporate the electrostatic protein environment and water solvent, respectively. The porphyrin distortions along the saddling, ruffling, and doming modes in the optimized structures are listed in Table 1. The equilibrium structure of isolated heme *b* was nearly the same as that of the Fe-porphyrin molecule, although it was slightly distorted along the saddling and ruffling modes (only in the result with a dielectric constant of 78.4; a distortion of 0 corresponds to the equilibrium structure of the Fe-porphyrin molecule). The magnitude of the ruffling distortion tends to increase from the five-coordinate state to the six-coordinate state, whereas that of the doming distortion has the opposite tendency. The optimized structures of each system are shown in Figure S2. These tendencies are consistent with a previous study that analyzed the distortion of Fe-porphyrin molecules, by Bikiel et al. [8]. The difference in the dielectric constant as a parameter of the PCM did not significantly affect the equilibrium structure of heme b.

### 3.2. Difference in Heme Distortions between Hb and Mb Homologs in the Oxy-State and Deoxy-State

**Figure 2:**
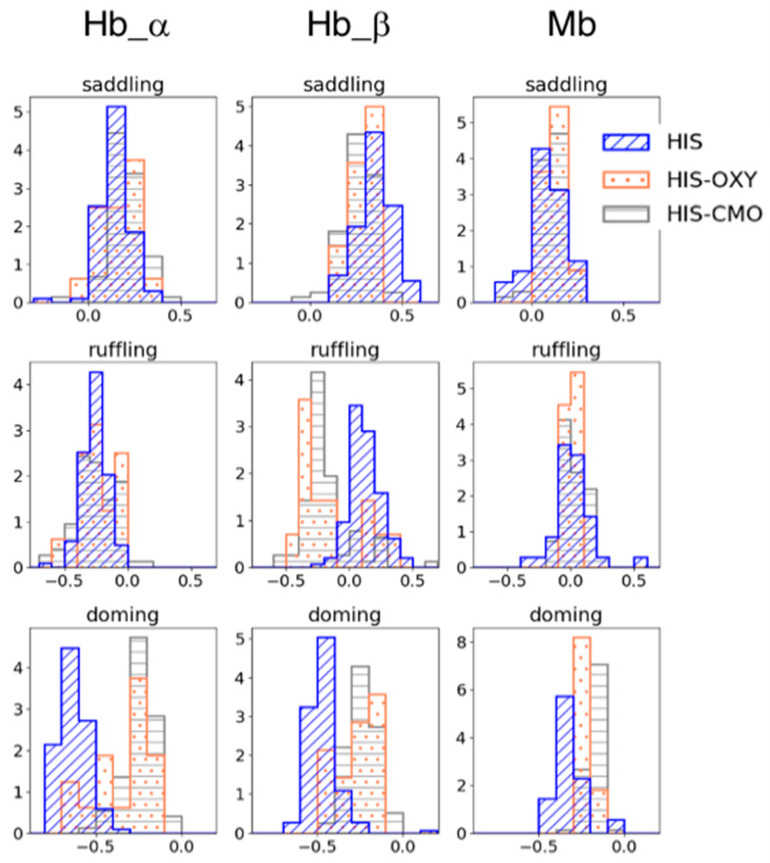
Porphyrin distortions of hemes in Hb subunits (the left two columns) and Mb (the right column). The blue with diagonal line represents the distortion in mono His-coordinated heme (HIS); the coral with dots in His-O_2_-coordinated heme (HIS-OXY); and the gray with horizontal stripes in His-CO-coordinated heme (HIS-CMO). The first, second, and third rows show the saddling, ruffling, and doming distortions, respectively.

To examine the effect of the protein pocket environment on heme distortion, we collated the structural data of Hb and Mb from PDB and analyzed the distribution of heme distortions with five-coordinate (mono-His coordinated) heme and six-coordinate heme, for which ligands are His-O_2_ (oxygen molecule) or His-CO (carbon monoxide). The homologous protein groups of the Hb α/β chain and Mb were obtained by clustering the amino acid sequences of heme proteins containing heme *b* or heme *c* using Cd-Hit with a threshold of 90% sequence similarity. Figure 2 shows the heme distortion along the saddling, ruffling, and doming modes in the Hb α/β chain and Mb. Whereas no significant difference was observed between the five-coordinated and six-coordinated hemes in the saddling and ruffling distortions, except for the Hb β chain, the doming distortion (absolute values) tended to decrease from the five-coordinate state to the six-coordinate state. This corresponds to the tendency observed in isolated heme *b* in Section 3.1.

In addition, a difference was observed in the doming distribution between the Hb α/β chain and Mb, even though they have similar functions (oxygen binding) and structure. The mean values of the doming distortion in each subunit in the His-coordinated and His-O_2_-coordinated states were −0.633 (HIS) and −0.354 (HIS-OXY) for the Hb α chain, −0.467 (HIS) and −0.264 (HIS-OXY) for the Hb β chain, and −0.321 (HIS) and −0.221 (HIS-OXY) for Mb. The change in doming distortion from the deoxygenated state to the oxygenated state in Mb was smaller than that in Hb. Since doming distortion is known to correlate with the oxygen affinity of heme, these differences in doming distortions could be involved in their protein function. Our previous study [12] suggested a correlation between the protein environment and heme distortion along saddling, ruffling, and doming modes. Considering these points, a protein environment might control the heme distortion, as it is appropriate to protein function. In the following, we focus on the doming distortion of heme molecules in Hb and Mb.

### 3.3. Stability of Protein Whole Structure and Conformation of Heme-Binding Pocket

We computed the RMSD values of Cα atoms for the whole protein structure and pocket residues to analyze the difference in structural stability between the deoxy-heme-bound, heme-unbound, and oxy-heme-bound proteins. Mean values and standard deviations are presented in Table 2. RMSD values were time-averaged over the 200 ns × 2 production run for each system for Hb, dMb, and oMb, and 200 ns × 3 for apoMb. For the Hb trajectories, RMSD values were averaged for each subunit (α or β chain). The entire protein structure was very stable in the deoxy-heme-bound and oxy-heme-bound proteins, and no significant difference was observed between them. In the apo (heme-unbound) protein, the RMSD values were larger than those in the holo (deoxy-heme-bound) state, but less than 2.0 Å. Compared to the RMSD values of the pocket residues (Table 2), the structural stability was high, particularly in the heme-binding pocket in the holo proteins (deoxy and oxy), and was low in the heme-binding pocket in the apo protein, suggesting that heme binding stabilizes the pocket conformation of its host protein. In the comparison between apoHb and apoMb, the standard deviation of RMSD was much larger, especially in the heme-binding pocket in Mb, than that in the Hb α/β chains. This indicated that the pocket conformation of Mb is more flexible than that of Hb. The time sequences of RMSD values for each trajectory were shown in Figure S3. Because the behavior of apoMb was different between two trajectories, we performed an additional simulation. The thermodynamic property (mean temperature and pressure) of each trajectory was presented in Table S1.

### 3.4. Fluctuations in Amino Acid Residues in the Heme-Binding Pocket

**Figure 3:**
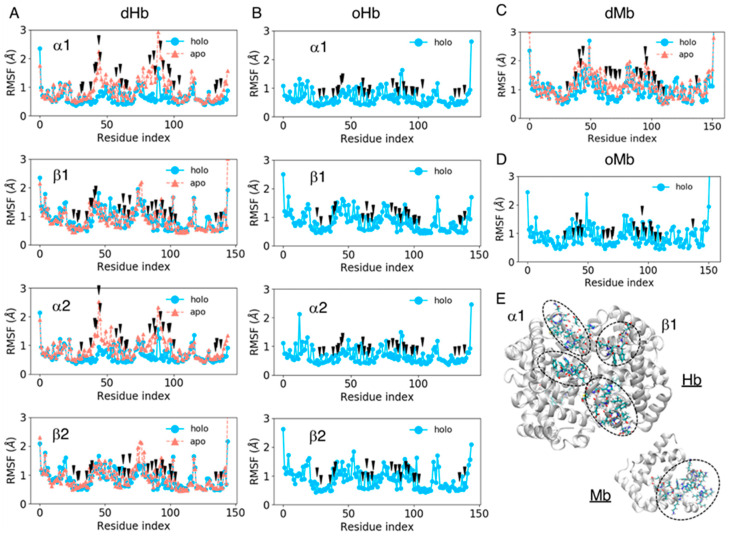
The root mean square deviation (RMSF) values of each residue averaged over the trajectories of the same system for dHb (**A**), oHb (**B**), dMb (**C**), and oMb (**D**). The sky-blue lines represent the holo (heme-bound) proteins, and the coral lines are apo proteins. The black arrows represent the pocket residues. (**E**) Protein structures of apoHb and apoMb. The backbone of each protein is shown as the white cartoon, and the amino acid residues of indices 40–45 and 75–90 and with indices of 40–50 and 60–70 for Hb and Mb, respectively, are represented by licorice model.

We computed the RMSF values of amino acid residues for all the systems considered to estimate the fluctuation of each residue, including the side chain atoms. The RMSF values for each residue were averaged over the trajectories of the same system and plotted for each chain as shown in Figure 3. The black arrows in Figure 3 represent pocket residues. The mean values and standard deviations of the RMSF pocket residues are shown in Table 3. The fluctuation in the pocket residues was very small in the holo (heme-bound) state (less than 0.9 Å) and became slightly larger in the apo state, at approximately 1.0 Å. The increase in the fluctuation amplitude was remarkable in the Hb α chain and Mb. As shown in Figure 3, the fluctuation of pocket residues became large especially in the regions of indices 40–45 and 75–90 for Hb α chains, while no large difference was observed between the apo and holo proteins for Hb β chains. For Mb, the regions with indices 40–50 and 60–70 became more flexible in the apo protein. Large fluctuations in the apo state might be because these regions were located in the entrance of pocket (Figure 3E). A stability in Hb β chain may be caused by the structural maintenance by ionic residues (sequence of residue indices 40–45 is FESFGD for Hb β chain, c.f. TYFPHF for Hb α chain). The flexibility in the regions 75–90 in Hb would be due to the absence of interaction between heme and ligand residue: The ligand residues in Hb α and β chains are indices 86 and 90, respectively.

**Figure 4:**
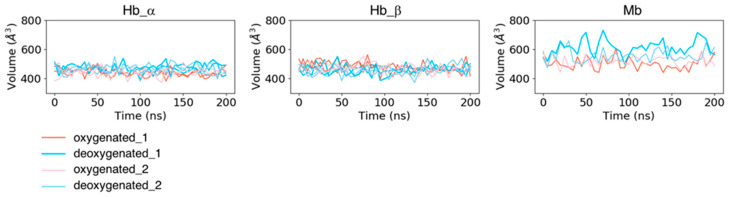
Time courses of volumes of heme-binding pocket for each subunit of Hb (the left two panels) and Mb (the right panel). The coral and pink lines represent the trajectories of proteins in the oxygenated state, and the sky-blue and light-blue lines are those in the deoxygenated state.

In addition to the fluctuations in each residue, we computed the time course of pocket volumes every 5 ns (Figure 4). No large difference was observed in pocket volumes from the initial state to the last state for all the systems. The mean values and standard deviations of the pocket volumes for each subunit averaged over two trajectories (and the same subunits for Hb) were 467.427 ± 31.661 Å^3^ for dHb α chains, 458.976 ± 31.867 Å^3^ for dHb β chains, 443.994 ± 26.768 Å^3^ for oHb α chains, 472.110 ± 33.784 Å^3^ for oHb β chains, 585.037 ± 55.945 Å^3^ for dMb, and 510.512 ± 38.262 Å^3^ for oMb. The mean values averaged over each trajectory are presented in Table S1. Mb had a larger absolute value of the pocket volume and fluctuation amplitude than Hb. Because the variances in RMSD values of the pocket residues in Mb in the holo (deoxy- and oxy-heme-bound) states were almost the same or smaller than those in the Hb β chain, this fluctuation of the pocket volume would be due to the fluctuations of the side chains of the pocket residues.

### 3.5. Estimation of the Effect of Protein Conformations on Heme Distortion

**Figure 5:**
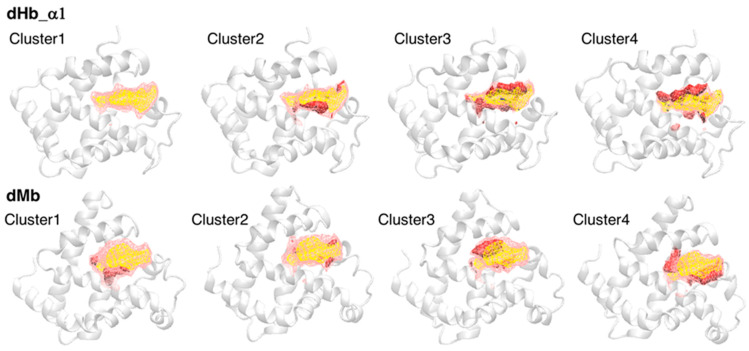
The average pocket structures of each cluster. The first and second rows show the structures for dHb α chain 1 and dMb, respectively. The yellow and pink regions drawn with the wireframe represent the pocket structures averaged over all samples (cluster 1 to 4) and over each cluster, respectively. The red and blue regions drawn with the solid surface indicate the increase and decrease from the average of all samples, respectively. The representative structures of each cluster are shown with white cartoon (backbone only).

Although our previous study suggested a correlation between heme distortion and the composition of amino acid residues around heme [12], protein dynamics must also affect the conformation (distortion) of heme. We estimated the doming distortion in representative pocket conformations in our simulation trajectories. First, we clustered the conformations of the heme-binding pocket to extract representative structures. Snapshots were extracted every 5 ns from each trajectory (41 frames per trajectory), and snapshots from two trajectories of the same system were combined (82 frames per system). These snapshots were divided into four clusters using a hierarchical clustering method, and four representative samples of the heme-binding pocket were selected using POVME 3.0. Table 4 lists the number of samples in each cluster. Most samples belonged to cluster 1 or cluster 2, implying that the distribution did not have characteristic clusters. The protein structures of representative samples and pocket structures averaged over each cluster or all clusters were shown in Figure 5 (only dHb a chain1 and dMb were shown, the others can be shown in Figure S4). As shown in Figure 5, conformational differences from the average pocket were in various regions, indicating a sampling of various pocket conformations.

Next, we optimized the representative conformations of each cluster obtained using POVME 3.0 software by using the ONIOM method (second model in Section 2.5) and computed the doming distortion of the optimized structures (the validity of the estimation of doming distortion by ONIOM is discussed in the next section). For two of the 40 clusters, the optimization of a representative structure did not converge, and another conformation in the same cluster was used (cluster 1 of dHb α2 and cluster 4 of oHb β1). For one of these clusters, optimization did not converge in the alternative structure (cluster 4 of oHb β1). The calculated doming distortions are presented in Table 5. The mean values over the clusters were the weighted means obtained by multiplying the sample number; for example, the mean for dHb a1 was calculated as (−1.058 × 71 − 0.825 × 7 − 0.962 × 2 − 0.689 × 2)/82. Comparing the doming distortions in Hb with those in Mb, we could see that heme molecules tend to be distorted along the doming mode more largely in Hb than in Mb, and the difference in the doming distortion between the oxygen-unbound (mono His-coordinated) state and oxygen-bound (His-O_2_-coordinated) state in Hb (Hb α chain) was larger than that in Mb (Hb β chain). These corresponded to the observations from the PDB data mentioned in Section 3.2. The magnitude of distortion was distributed over a wide range in each system, for example, −1.136 to −0.352 for the dHb α chain, and −0.760 to −0.129 for dMb. The physical property of proteins such as an oxygen affinity would appear as an average of physical property of heme with different magnitudes of distortion.

**Figure 6:**
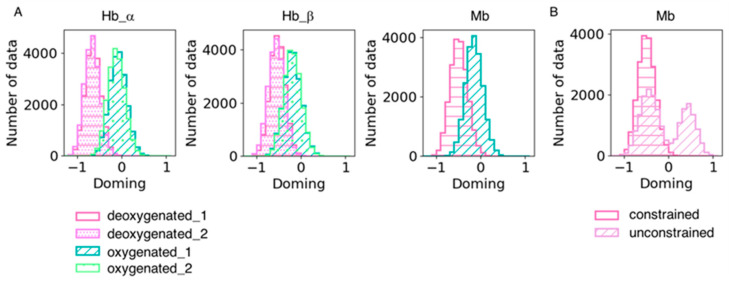
(**A**) Doming distortions in each subunit in the simulation trajectories. Two trajectories of the same system were combined. The hot pink with horizontal stripe and the violet with dots represent the deoxygenated state, and the sage green with a diagonal line and pale green with rough dots are the oxygenated state. (**B**) Comparison of doming distortions in the simulation trajectories with and without constraint between heme and its ligand His. The hot pink with a horizontal stripe and the plum with a diagonal line represent the dMb trajectories with and without constraint, respectively.

We also analyzed heme distortion in the simulation trajectories. Snapshots were sampled every 20 ps, and histograms were generated for the doming distortion (Figure 6). As shown in Table 6, the doming distortion of each system qualitatively corresponded to the results obtained from the QM calculations. Hemes tend to be less distorted along the doming mode in dMb than dHb under the same force field of the heme molecule, suggesting an effect of the protein environment on heme distortion. As mentioned in the Methods section, we constrained the distance between heme and its ligand histidine in the dMb and oMb simulations because coordination could not be maintained without a constraint. This might be caused by the difference in heme conformation between Mb and Hb; the force field of heme used in this study was optimized for heme in hemoglobin and was unsuitable for simulations of heme in Mb. The doming distribution in the unconstrained dMb trajectories is shown in Figure 6B. The distribution was bimodal and showed an inversion of the distortion. It is possible that the conformation of hemes is affected by its binding pocket environment and is important for maintaining coordination with its host protein.

### 3.6. Validation for an Estimation of Doming Distortion with the ONIOM Method

**Figure 7:**
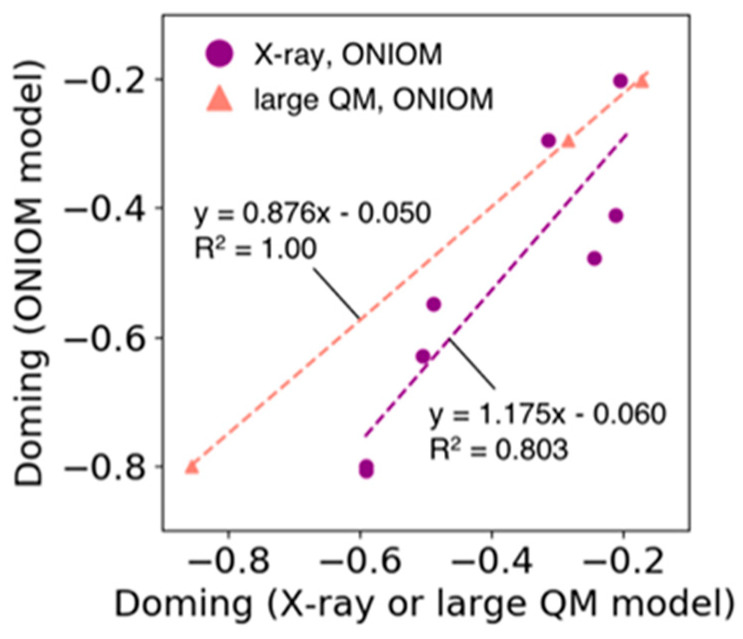
Plot of the doming distortion calculated based on the ONIOM model versus that based on the X-ray structure (filled circle colored in purple) and the large QM model (filled triangle colored in salmon). The horizontal axis represents the doming values calculated from X-ray structures or computed based on the large QM model. The vertical axis shows the doming values calculated based on the ONIOM model. The dotted lines represent the regression line for each group (line colors correspond to the colors used in the scatter plot).

In the ONIOM model used in Section 3.5, the high layer consists only of heme and its ligands, and all the atoms in the low layer are fixed during optimization, allowing an efficient estimation of the doming distortion of the heme molecule. Figure 7 shows the correlation between the doming distortions observed in the X-ray structures of Hb and Mb and the distortions estimated by the ONIOM model (purple line in Figure 7, R^2^ = 0.80). In addition, we compared the results of the ONIOM model with those from the calculations of larger QM model systems. The latter model system consists of heme and adjacent amino acid residues for which the heavy atoms are within 4.5 Å from the Fe-porphyrin skeleton of the heme molecule. Only the heme and hydrogen atoms were optimized. The results are shown in Figure 7 as points and lines with salmon color (we examined only three systems because this process is time-consuming). The doming values calculated using the former ONIOM method corresponded well with those obtained from the latter large QM model (regression coefficient = 0.9, R^2^ = 1.0). From these results, we determined that the estimation of doming distortion using the ONIOM model was appropriate.

## 4. Conclusions

In this study, we investigated fluctuations in the conformation of the heme-binding pocket and heme distortion for Hb and Mb to elucidate the effect of the protein environment on the distortion of heme porphyrin. First, to estimate the effect of ligand(s) on heme distortion, we optimized the model systems of isolated heme by performing QM calculations. The calculated distortion of the optimized structures showed a tendency similar to that of the Fe-porphyrin molecule reported by Bikiel et al. [8]. We compared the heme distortion between isolated heme and hemes in Hb and Mb from PDB data. This comparison indicated that the protein environment distorts heme in the doming mode more than the isolated ligand(s), especially for proteins in the deoxygenated state. The difference in doming distortion between the deoxygenated and oxygenated states in Hb was larger than that in Mb. We focused on doming distortion because it correlates with the oxygen affinity of heme.

Next, we computed the fluctuation of the heme-binding pocket and heme distortion in its host protein for Hb and Mb using MD simulations to clarify the relationship between the protein environment and heme distortion. The trajectory analysis of the simulations suggested that the protein environment is more flexible for Mb than for Hb. Hierarchal clustering was then performed for the structures of each protein subunit sampled based on MD simulations, and the doming distortion was estimated for the representative conformation of each cluster using the ONIOM model. The results showed the same tendency as the survey of the PDB data; specifically, heme tended to be distorted along the doming mode more largely in the dHb than in the dMb, and the difference in the distortion between the deoxygenated and oxygenated states was larger in Hb than in Mb and was different even between the Hb α chain and β chain. These differences might be related to differences in function between Hb and Mb. A previous study by Bringas et al. [18] showed a detailed difference in the mechanism underlying the affinity change between Hb α and β chains. It is reasonable that a difference in the doming distortion of heme was observed between Hb α and β chains.

We also calculated the doming distortion of heme from the MD trajectories and showed that the classical MD simulation can reproduce the distribution of heme distortion obtained by QM calculations. Finally, we validated the ONIOM model used in this study by comparing the doming values obtained from the X-ray structure and those calculated using our ONIOM model. A high correlation was observed between them. Although this method should be examined for other proteins, it could be possible to refine the heme structures obtained from X-ray crystallographic studies using our method. We believe that our finding helps better understand the structure–function relationship in heme proteins and will enable functional design of heme protein in the future.

## Figures and Tables

**Table 1:** Porphyrin distortions in heme *b*.

System	Dielectric Constant	Saddling [Å]	Ruffling [Å]	Doming [Å]
heme *b*	4.0	−0.133	−0.001	−0.004
His-coordinated heme *b*	4.0	−0.150	−0.040	−0.115
His-O_2_-coordinated heme *b*	4.0	−0.065	−0.642	0.069
heme *b*	78.4	−0.136	0.105	−0.004
His-coordinated heme *b*	78.4	0.005	0.034	−0.138
His-O_2_-coordinated heme *b*	78.4	−0.043	−0.393	0.016

**Table 2:** The mean values and standard deviation of root mean square deviation (RMSD) values of the whole protein structure and heme-binding pocket (in parentheses).

System	RMSD (deoxy) [Å]	RMSD (apo) [Å]	RMSD (oxy) [Å]
Hb α chain	0.716 ± 0.084 (0.541 ± 0.090)	1.079 ± 0.217 (1.276 ± 0.310)	0.716 ± 0.080 (0.537 ± 0.074)
Hb β chain	0.998 ± 0.179 (0.705 ± 0.179)	1.702 ± 0.197 (1.901 ± 0.248)	0.989 ± 0.119 (0.664 ± 0.101)
Mb	1.076 ± 0.145 (0.726 ± 0.141)	1.896 ± 0.576 (2.040 ± 0.746)	0.941 ± 0.196 (0.559 ± 0.103)

**Table 3:** The mean values and standard deviations of root mean square deviation (RMSF) values of the pocket residues.

System	RMSF (deoxy) [Å]	RMSF (apo) [Å]	RMSF (oxy) [Å]
Hb α chain	0.558 ± 0.180	0.913 ± 0.461	0.670 ± 0.454
Hb β chain	0.692 ± 0.172	0.780 ± 0.209	0.699 ± 0.179
Mb	0.849 ± 0.308	1.190 ± 0446	0.720 ± 0.224

**Table 4:** The number of members in each cluster.

System	Cluster 1	Cluster 2	Cluster 3	Cluster 4
dHb α1	71	7	2	2
dHb α2	54	21	6	1
dHb β1	77	3	1	1
dHb β2	66	8	6	2
oHb α1	72	5	4	1
oHb α2	72	5	4	1
oHb β1	72	5	3	2
oHb β2	71	5	3	3
dMb	38	27	11	6
oMb	59	17	5	1

**Table 5:** The doming distortions estimated for the representative structures of each cluster.

System	Cluster 1	Cluster 2	Cluster 3	Cluster 4	Mean (Clusters)	Mean (Subunits)
dHb α1	−1.058	−0.825	−0.962	−0.689	−1.027	−0.966
dHb α2	−1.136	−0.961	−0.352	−0.852	−1.030	
dHb β1	−0.904	−0.815	−0.828	−0.647	−0.897	
dHb β2	−0.905	−0.865	−0.981	−0.961	−0.908	
oHb α1	−0.517	−0.665	−0.408	−0.451	−0.520	−0.595
oHb α2	−0.430	−0.278	−0.417	0.026	−0.415	
oHb β1	−0.817	−0.485	−0.446	-	−0.773	
oHb β2	−0.702	−0.476	−0.520	−0.455	−0.672	
dMb	−0.760	−0.364	−0.431	−0.129	−0.540	-
oMb	−0.422	−0.422	0.212	−0.559	−0.385	-

**Table 6:** The mean values and standard deviation (std) of the doming distortions for each subunit in each simulation trajectory. The mean values over four subunits were also calculated for dHb and oHb (right column).

System	Mean ± std	Mean (Subunits)
dHb α1	−0.645 ± 0.173	−0.601
dHb α2	−0.666 ± 0.173	
dHb β1	−0.530 ± 0.171	
dHb β2	−0.561 ± 0.178	
oHb α1	−0.085 ± 0.192	−0.150
oHb α2	−0.125 ± 0.188	
oHb β1	−0.195 ± 0.198	
oHb β2	−0.194 ± 0.194	
dMb	−0.491 ± 0.199	-
oMb	−0.148 ± 0.194	-

## Data Availability

The atomic coordinates of the heme proteins were downloaded from PDBj: https://pdbj.org/. Our collated data of hemes is available in PyDISH: https://pydish.bio.info.hiroshima-cu.ac.jp/ (accessed on 6 April 2021). For convenience, the list of PDB IDs is given in the Supplementary Materials.

## Supplementary Materials

The following supporting information can be downloaded at: https://www.mdpi.com/article/10.3390/life12020210/s1, List of PDB IDs, Figure S1: Illustration of QM models used in this study, Figure S2: The optimized structures of each system, Figure S3: The time sequence of RMSD values of the entire protein structure, Figure S4: The average pocket structures of each cluster. Table S1: Thermodynamic property of each trajectory, Table S2: Mean values and standard deviation of the pocket volume in each subunit in each trajectory.
