# Supplementary material for: Analysis of Fluctuation in the Heme-Binding Pocket and Heme Distortion in Hemoglobin and Myoglobin
Source: Life (Basel). 2022 Jan 29;12(2):210. doi: 10.3390/life12020210 (PMC8880375; doi:10.3390/life12020210)
Supplement: Supplementary file 1 [file life-12-00210-s001.zip › SI_revised.pdf]

Analysis of fluctuation in the heme-binding pocket and heme distortion in hemoglobin and myoglobin

Hiroko X. Kondo [1,2,3,*], and Yu Takano [2]

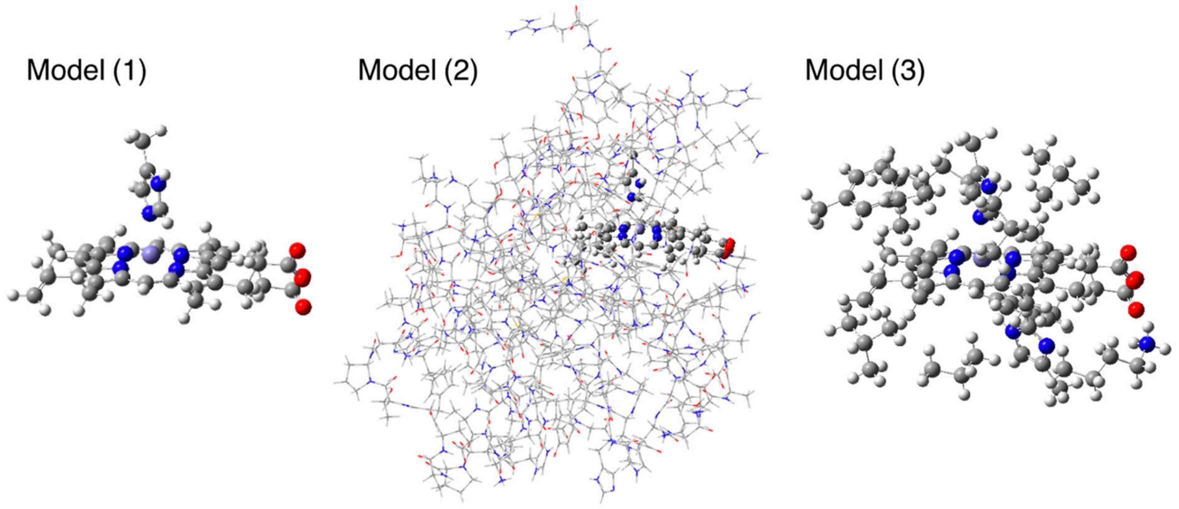

Figure S1. Illustration of QM models used in this study. All pictures were depicted using Hb a chain as an example. The ball and stick model and line model represent the high layer (QM: PBE0/6-31G(d)) and low layer (molecular mechanics: AMBER force field), respectively.

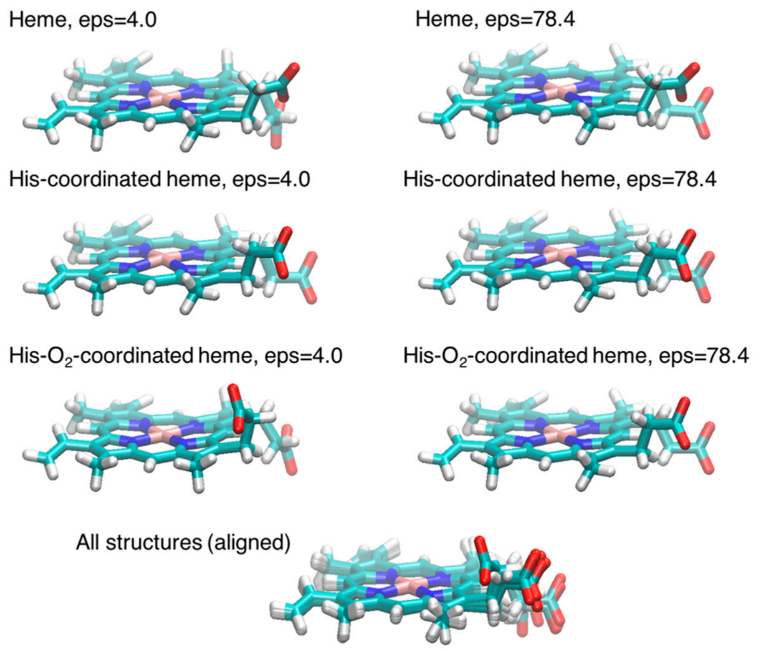

Figure S2. The optimized structures of each system. The 1st, 2nd, and 3rd lines represent the systems of heme *b*, His-coordinated heme *b*, and His-O_2-coordinated heme *b*, respectively, and "eps" means dielectric constant. The bottom line displays all above structures in a figure.

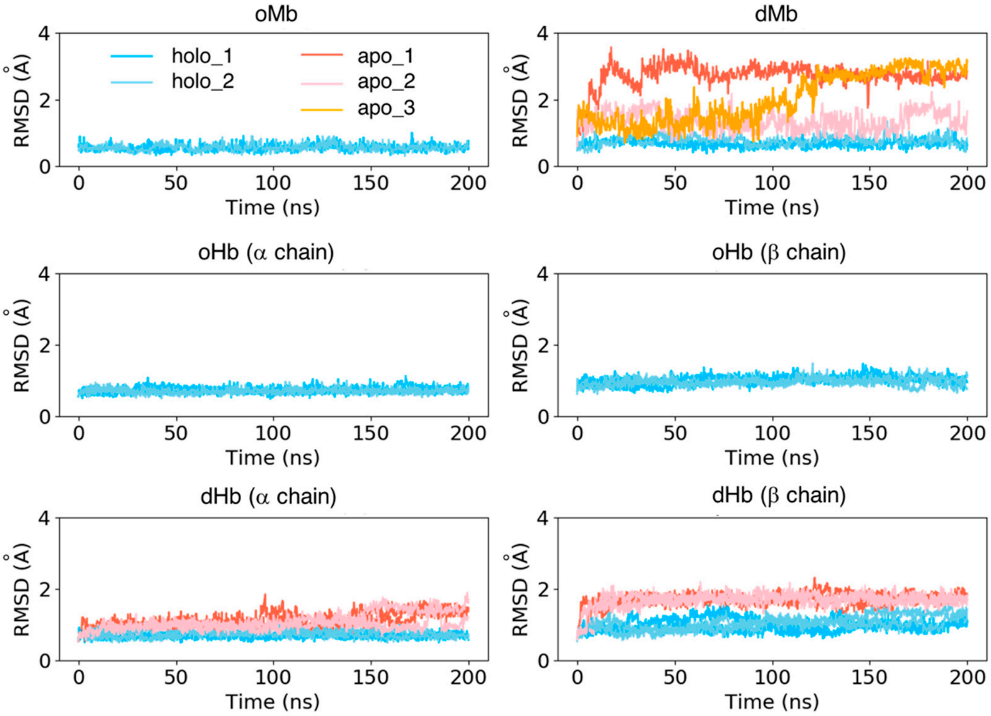

Figure S3. The time sequence of RMSD values of the entire protein structure. The deep sky-blue and sky-blue lines represent the 1st and 2nd trajectories of the holo (heme-bound) protein, respectively, The tomato, pink, orange lines are the 1st, 2nd, and 3rd (only for apoMb) trajectories of the apo (heme-unbound) protein, respectively. Two subunits are drawn with the same color for Hb a/b chain.

Table S1. Thermodynamic property of each trajectory. The values of temperature and pressure averaged over each trajectory were listed.

System	Temperature [K]	Pressure [bar]
dHb 1	300.0	1.01
dHb 2	300.0	1.14
apoHb 1	300.0	1.04
apoHb 2	300.0	1.11
oHb 1	300.0	0.98
oHb 2	300.0	1.05
dMb 1	300.0	1.20
dMb 2	300.0	1.08
apoMb 1	300.0	1.00
apoMb 2	300.0	1.20
oMb 1	300.0	1.15
oMb 2	300.0	1.24

Table S2. The mean values and standard deviation (std) of the pocket volume in each subunit in each trajectory.

System	Mean ± std [Å^3] (trajectory1)	Mean ± std [Å^3] (trajectory2)
dHb 1	466.073 ± 25.613	457.488 ± 33.651
dHb 2	475.049 ± 30.022	471.098 ± 29.834
dHb 1	462.927 ± 27.394	456.854 ± 33.004
dHb 2	460.683 ± 34.487	454.707 ± 31.314
oHb 1	446.463 ± 25.613	452.951 ± 23.947
oHb 2	438.732 ± 24.433	437.829 ± 29.794
oHb 1	477.585 ± 39.005	458.561 ± 29.228
oHb 2	478.244 ± 30.544	474.049 ± 31.654
dMb	613.049 ± 52.655	557.024 ± 43.794
oMb	502.732 ± 40.792	518.293 ± 33.807

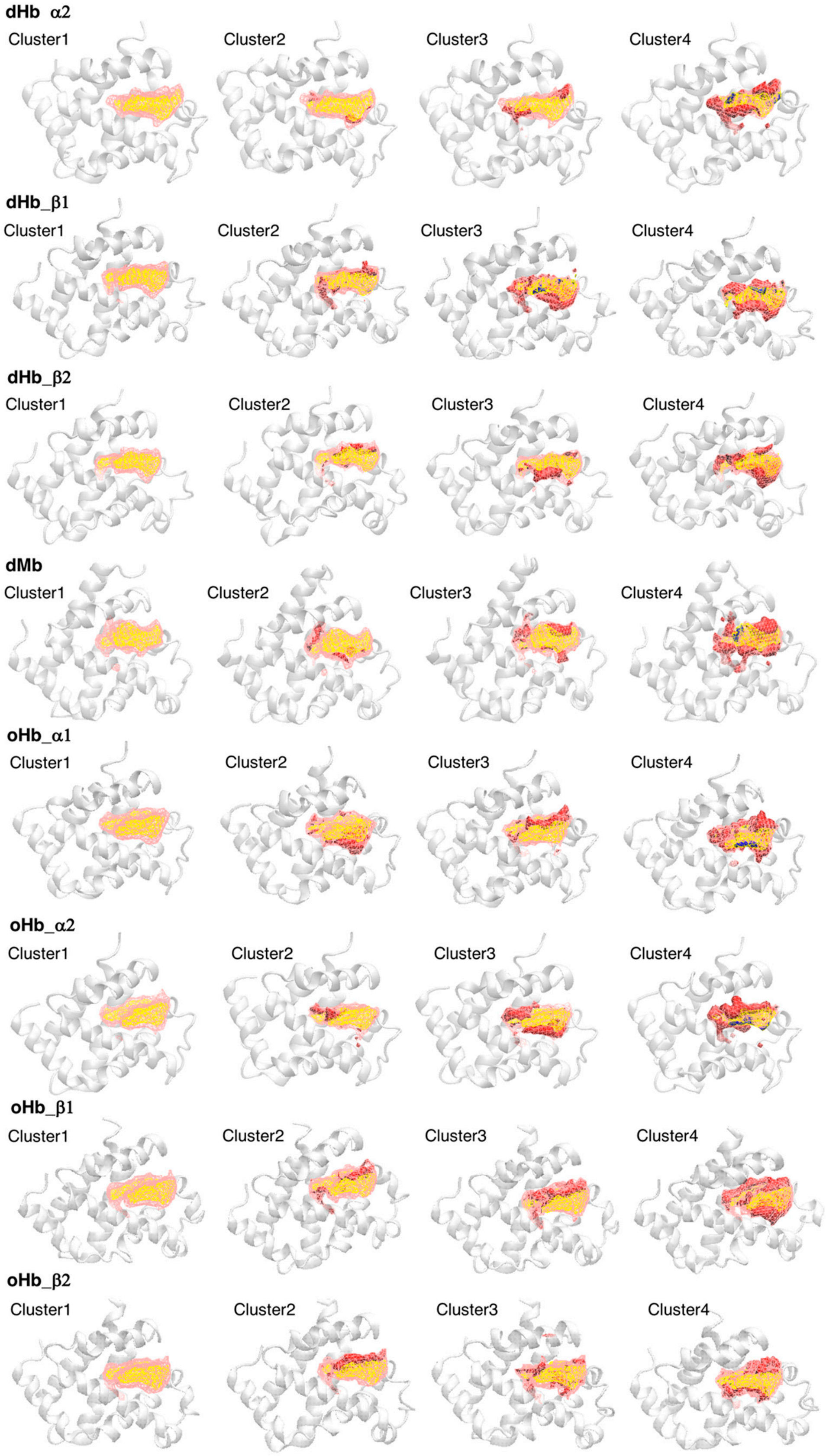
dHb α2
Cluster1
Cluster2
Cluster3
Cluster4
dHb_β1
Cluster1
Cluster2
Cluster3
Cluster4
dHb_β2
Cluster1
Cluster2
Cluster3
Cluster4
dMb
Cluster1
Cluster2
Cluster3
Cluster4
oHb_α1
Cluster1
Cluster2
Cluster3
Cluster4
oHb_α2
Cluster1
Cluster2
Cluster3
Cluster4
oHb_β1
Cluster1
Cluster2
Cluster3
Cluster4
oHb_β2
Cluster1
Cluster2
Cluster3
Cluster4

Figure S4. The average pocket structures of each cluster. The 1st to 8th rows show the structures for dHb a chain 2, dHb b chain 1, dHb chain 2, oMb, oHb chain 1, oHb chain2, oHb chain 1, and oHb chain 2, respectively. The yellow and pink regions drawn with the wireframe represent the pocket structures averaged over all samples (cluster 1 to 4) and over each cluster, respectively. The red and blue regions drawn with the solid surface indicate the increase and decrease from the average of all samples, respectively. The representative structures of each cluster were shown with white cartoon (backbone only).
